# Verix: a toolkit for benchmarking and harmonization of complex structural variants

**DOI:** 10.1093/bioinformatics/btag803

**Published:** 2026-10-29

**Authors:** Chris Rohlicek, Enzo Battistella, Anant Maheshwari, Tony Cui, Bert Huang, Yueyao Gao, Victoria Popic

**Affiliations:** Broad Institute of MIT and Harvard, Cambridge, MA 02142, United States; Broad Institute of MIT and Harvard, Cambridge, MA 02142, United States; Broad Institute of MIT and Harvard, Cambridge, MA 02142, United States; Broad Institute of MIT and Harvard, Cambridge, MA 02142, United States; Broad Institute of MIT and Harvard, Cambridge, MA 02142, United States; Broad Institute of MIT and Harvard, Cambridge, MA 02142, United States; Broad Institute of MIT and Harvard, Cambridge, MA 02142, United States

## Abstract

**Summary:**

Complex structural variants (CSVs) represent a critical yet under-characterized class of large-scale genomic rearrangements. CSV discovery requires not only the accurate detection of individual breakpoints but also their assembly into a single, structurally resolved CSV event. However, CSV method development is currently limited by a diagnostic gap: existing SV evaluation frameworks are designed for single-interval or single-junction SVs and cannot quantify multi-breakpoint CSV reconstruction fidelity or systematically distinguish between different types of reconstruction errors, such as over-segmentation or collapse of independent breakpoints. Here we present Verix, a comprehensive toolkit designed for the fine-grained evaluation and integration of CSVs. Verix introduces granular diagnostic metrics that enable researchers to thoroughly characterize CSV predictions and stratify performance based on breakpoint sensitivity, precision, and structural integrity. In addition to pairwise callset-to-truthset comparisons, Verix also provides an intra- and inter-callset harmonization module for generating consensus calls for a single tool’s output or across disparate discovery pipelines. As such, Verix provides the infrastructure needed to rigorously evaluate and reconcile CSV calls, accelerating the standardization of CSV discovery in both research and clinical settings.

**Availability and implementation:**

Verix is available under the MIT license at https://github.com/PopicLab/verix and https://doi.org/10.5281/zenodo.22998063.

## 1 Introduction

Complex structural variants (CSVs) are large-scale genomic rearrangements in which multiple breakpoints arise from a single mutational event, often involving combinations of inversions, deletions, and duplications of several genome segments. These range from localized events, such as deletion-flanked inversions and inverted duplications, to catastrophic chromosome-level shattering events, such as chromothripsis. Despite their critical role in the etiology of cancer and a wide array of common and rare diseases (Mantere *et al.* 2019, Collins *et al.* 2020, Li *et al.* 2020, Cosenza *et al.* 2022, Ek *et al.* 2026), CSVs remain substantially less characterized than simple structural variants (SVs) (Jung *et al.* 2025). This disparity is driven in large part by the methodological challenges inherent in CSV detection and evaluation. While the past decade has seen a rising number of CSV callers (Sedlazeck *et al.* 2018; Heller and Vingron 2019; Soylev *et al.* 2019; Lin *et al.* 2022a; 2022b; Popic *et al.* 2023; Smolka *et al.* 2024; Keskus *et al.* 2026), the lack of robust standards for CSV evaluation continues to hinder both method development and clinical adoption.

Existing standard SV benchmarking tools (English *et al.* 2022) have been designed for simple variants and typically evaluate predicted breakpoints or individual genome intervals in isolation, making them poorly suited for characterizing the accuracy of multi-breakpoint CSVs, which require not only accurate breakpoint detection but also their correct assembly into a single CSV event. In practice, CSV predictions may vary in completeness and breakpoint accuracy. For instance, a caller may only partially reconstruct a CSV event (i.e. report a subset of its breakpoints), fragment a single CSV into multiple independent calls (over-segmentation), erroneously aggregate unrelated events into a single record (over-aggregation), or introduce spurious breakpoints due to alignment artifacts. In the absence of CSV-aware benchmarking frameworks, current CSV studies have relied on ad hoc evaluation strategies—matching breakpoints, junctions, or segments independently against the truthset (Lin *et al.* 2022b, Popic *et al.* 2023, Keskus *et al.* 2026), matching the maximal interval defined by a CSV’s flanking breakends (Lin *et al.* 2022a; 2022b); or jointly matching CSV segments (Lin *et al.* 2022b). However, these strategies do not provide stratified, fine-grained diagnostics of CSV reconstruction.

Beyond truthset-based benchmarking, downstream CSV analysis often requires the consolidation of predicted calls into a harmonized consensus callset. Whether arising from a single algorithm or a multi-caller ensemble pipeline, SV discovery workflows frequently produce near-duplicate calls. Such redundancies may stem from alignment ambiguity in repetitive genomic contexts, alignment artifacts, or divergent interpretations of the same SV signature. To streamline downstream analysis, such calls must be collapsed into a single record. However, existing consensus tools (Jeffares *et al.* 2017, English *et al.* 2022, Kirsche *et al.* 2023) operate on individual intervals, whereas collapsing CSVs requires joint multi-breakpoint comparisons. Furthermore, in multi-caller pipelines, consensus generation may additionally require support for multiple CSV reporting formats and naming conventions.

To address these infrastructure gaps, we developed Verix, a toolkit designed to provide comprehensive diagnostics and harmonization of CSV callsets. At a high level, Verix defines CSV evaluation and integration as a structural reconstruction problem, offering granular metrics for performance assessment. Verix provides the following key features:

- *Optimal multi-breakpoint CSV matching*: a matching engine that identifies the best correspondence between the breakpoints (genomic coordinates) of a predicted CSV and the ground truth.
- *Reconstruction fidelity metrics*: stratified classification and scoring based on breakpoint sensitivity, structural integrity, and breakpoint precision, which distinguishes between full reconstruction, partial capture, over-aggregation, and fully spurious calls.
- *Detailed callset annotation*: comprehensive reporting of all candidate matches and their match quality (breakpoint composition and proximity) for every predicted record.
- *Visual analytics*: automated diagnostic plots summarizing performance.
- *Consensus generation*: an intra- and inter-callset CSV collapse engine, which reconciles near-duplicate records based on their breakpoint composition.
- *Flexible format support*: ingestion of several CSV reporting formats, as well as minimal format requirements to streamline compatibility with custom pipelines.

By providing a CSV-oriented toolkit, Verix bridges the gap between raw breakpoint detection and structural resolution, offering a foundation for the standardized evaluation of complex genomic rearrangements.

## 2 Methods

### 2.1 CSV benchmarking

To compare two callsets—namely, a predicted set of calls (query) and a ground-truth set (target), Verix first parses their corresponding input VCF files to aggregate records corresponding to the same CSV (e.g. all records linked by a unique identifier). Each assembled CSV event is then represented by the three-tuple (I,B,T), where *I* represents the unique identifier of this CSV (either provided in the input or assigned by Verix), $B=(b_{1},b_{2},\ldots ,b_{n})$ is the set of all genomic coordinates involved in the CSV (i.e. all its induced breakpoint positions—which may span one or multiple chromosomes—grouped by chromosome and ordered by position), and *T* is the aggregate SV type (the set of type labels collected from each linked record). Since breakpoints are grouped and evaluated by their specific genomic coordinates, this representation natively supports both intra- and inter-chromosomal events (e.g. translocations).

For every query CSV call *P*, matching is performed in three main stages: (1) ground-truth candidate identification, (2) optimal match assignment, and (3) match classification. To identify candidate matches in the truthset, Verix considers a ground-truth event *G* to be a valid candidate if there exists at least one pair of concordant breakpoints ($b_{p}\in B_{P},b_{g}\in B_{G}$) such that both breakpoints are on the same chromosome and their genomic distance $d(b_{p},b_{g})\leq \delta$, where δ is the user-specified threshold for the maximum distance allowed for a breakpoint match. In stage (2), Verix computes the joint breakpoint alignment between *P* and all the identified candidates to select the optimal match $G_{\mathrm{opt}}$. This task is formulated as minimum-weight maximum-cardinality bipartite matching between two breakpoint sets that (1) maximizes the number of matched breakpoint pairs $|B_{P}\cap B_{G}{|}_{\delta }$ and (2) minimizes the cumulative distance $\sum d(b_{p},b_{g})$ across all breakpoint matches (Fig. 1A). The optimal alignment is computed for each candidate and the candidate with the highest number of matched breakpoints and minimal breakpoint distance is assigned as the optimal match. Note: by default, Verix operates on breakpoints (genomic coordinates) to maximize cross-caller compatibility; however, the matching engine can optionally be configured to operate on breakends instead (which requires an agreement on both position and orientation) and/or to treat novel sequence insertion breakpoints as a distinct breakpoint type, matched only to other insertion breakpoints. Finally, in stage (3) the call *P* is assigned one of the following primary classifications based on its optimal match and the global context of all matches (Fig. 1B):

**Figure 1:**
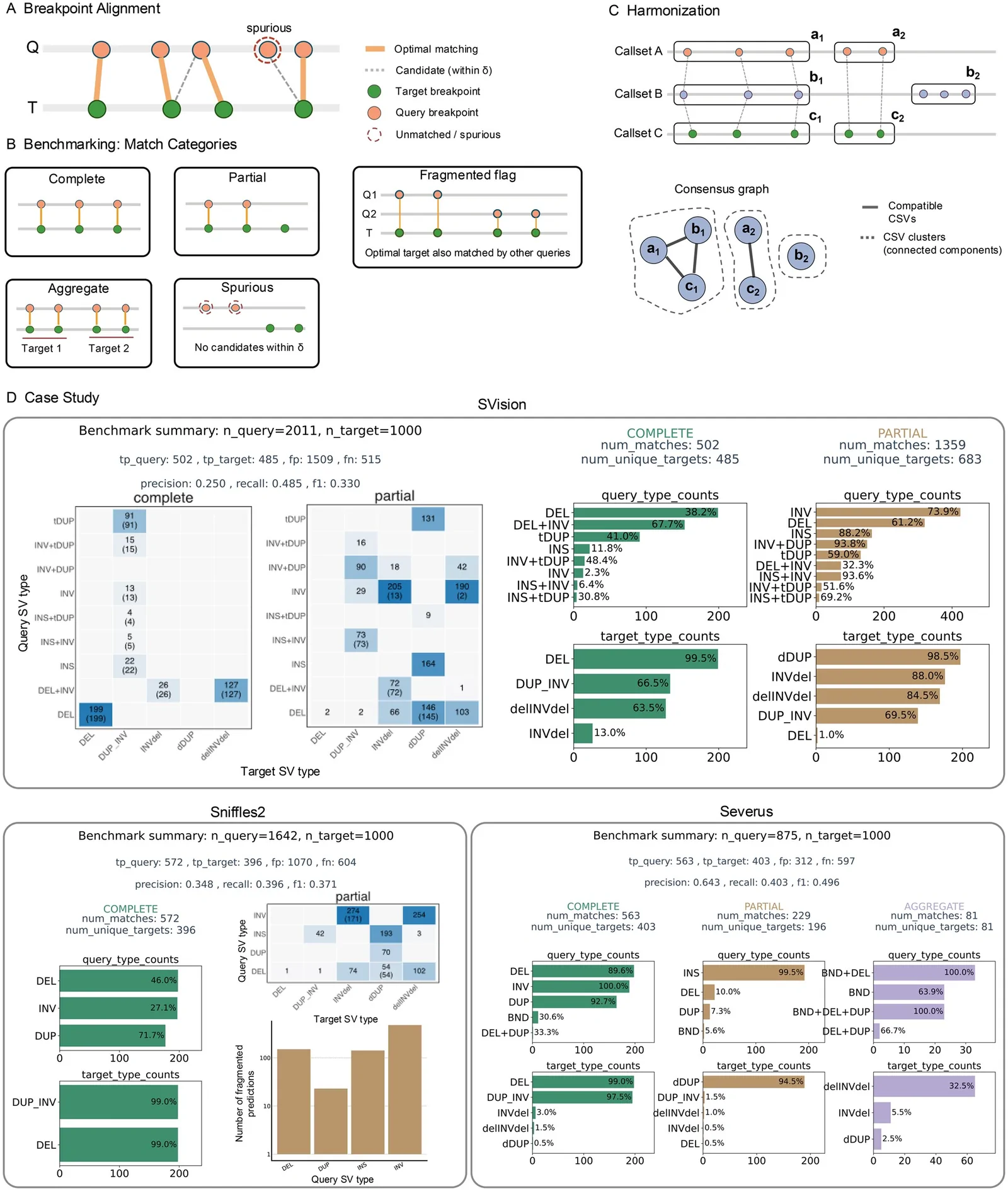
Overview of Verix. A. (A) Predicted CSV (Q) is aligned to a ground-truth CSV (T) by treating their breakpoints as a bipartite graph, where edges connect every pair of breakpoints within a user-specified distance threshold δ (dotted), and the optimal alignment is computed as the minimum-weight maximum-cardinality matching over these edges (solid). (B) Each predicted CSV is assigned to one of four mutually exclusive match categories based on its optimal alignment to the ground truth. *Complete*: every query breakpoint is matched to a distinct target breakpoint of the same event; *Partial*: only a subset of query or target breakpoints are aligned; *Aggregate*: distinct subsets of the query’s breakpoints align to multiple distinct target events (Target 1 and Target 2); *Spurious*: no query breakpoint has any candidate match in the truthset. The fragmented flag (right) marks calls whose optimal target (T) is also covered by other query records (Q1 and Q2). (C) Near-duplicate CSVs are merged across multiple callsets (A, B, C). CSVs from each input callset ($a_{1},a_{2},b_{1},b_{2},c_{1},c_{2}$) are first aligned pairwise using the same multi-breakpoint matching engine; CSVs are then converted to nodes in a consensus graph, with edges connecting any two events whose breakpoints are compatible. Connected components in this graph correspond to the consensus CSV clusters: $\left(a_{1},b_{1},c_{1}\right)$, $\left(a_{2},c_{2}\right)$, and $\left(b_{2}\right)$ are merged into three consensus events, respectively. (D) Snapshot of Verix outputs generated for three long-read SV callers (SVision, Sniffles2, and Severus) benchmarked against a simulated truthset of 1000 SVs of five different types. Summary statistics are shown for each caller, along with a subset of other reported plots (e.g. the predicted-versus-true SV type correspondence heatmap faceted by match category).

- *spurious* iff no candidate matches were found
- *complete* iff $|B_{P}|=|B_{G_{\mathrm{opt}}}|=|B_{P}\cap B_{G_{\mathrm{opt}}}{|}_{\delta }$
- *aggregate* iff $\exists b_{p}\in B_{P}\setminus B_{G_{\mathrm{opt}}}$ s.t. $b_{p}$ is a match to a different ground-truth event
- *partial* otherwise.

Ultimately, the results of the evaluation are written to a VCF file, where each query CSV is annotated with its assigned match classification and detailed information about $G_{\mathrm{opt}}$ and all other explored candidate matches. In particular, for each ground-truth candidate event *G*, Verix appends a 4-tuple match record (N,M,C,Z), where *N* indicates how many breakpoints were matched, *M* lists all the matched breakpoints and their distance, *C* indicates whether the match was *full* (all breakpoints of $B_{G}$ were matched) or *partial*, and *Z* flags if the matched breakpoints were consecutive in each chromosome. To further stratify reconstruction error types, Verix applies two diagnostic tags: a *spurious* tag is added to annotate how many breakpoints in an aggregate or partial call did not match any ground-truth event, and a *fragmented* flag is added to an event if there is at least one breakpoint in its optimal match that was captured by other calls but not by this event.

#### 2.1.1 Performance metrics

Verix aggregates CSV-level classification results and annotations to generate a comprehensive set of benchmarking metrics and plots. To assess whole CSV detection, Verix provides standard precision, recall, and F1 scores, where a true positive (TP) is defined as a complete match between a query call and a ground-truth event. Since multiple query calls may fully match the same ground-truth event, we distinguish $TP_{\mathrm{query}}$ (the number of query calls with a complete match) from $TP_{\mathrm{target}}$ (the number of ground-truth events with at least one complete match). A false positive (FP) is any query CSV which is not a TP, and a false negative (FN) is a ground-truth event that does not have a TP match. As such, precision is computed as the ratio of true positive query calls to the total number of predictions ($\mathrm{precision}=\frac{TP_{\mathrm{query}}}{TP_{\mathrm{query}}\;+\;FP}$); recall is computed as the ratio of true positive target events to the total number of ground-truth events ($\mathrm{recall}=\frac{TP_{\mathrm{target}}}{TP_{\mathrm{target}}\;+\;FN}$); and the F1 score is given by $F1=2\times \frac{\mathrm{precision}\;\times \;\mathrm{recall}}{\mathrm{precision}\;+\;\mathrm{recall}}$. Given the lack of a consistent CSV naming convention, Verix does not enforce CSV types to match by default; however, it provides a parameter to enforce strict type reporting, which would require a call to match both the breakpoints and the type of a ground-truth CSV to be considered a true positive.

Beyond strict CSV recovery metrics, Verix also quantifies partial recovery statistics and other performance indicators to provide nuanced diagnostics. In particular, it calculates the proportion of CSVs assigned to each match category, allowing method developers to identify specific breakpoint grouping errors. For example, a large number of aggregate calls may signal over clustering, while many partial calls may indicate over-segmentation.

For each match classification, Verix also outputs the following: (i) mean breakpoint distance: measures how much the predicted breakpoints deviate from the ground truth (computed for optimal matches only), (ii) mean breakpoint hit rate: measures how many breakpoints were matched in the optimal match, (ii) mean spurious breakpoint rate: measures how many extra spurious breakpoints were predicted per query, and (iv) average number of ground-truth events matched by a single record.

All performance metrics are reported as a JSON file to facilitate downstream evaluation. Additionally, Verix generates a PDF report with various diagnostic plots summarizing how query events aligned to the target, including breakpoint-level accuracy and coverage, the prevalence of spurious and fragmented calls, and the correspondence between query and target SV types—stratified by SV type and match category.

### 2.2 CSV harmonization

Verix can be used to merge near-duplicate CSVs within a single callset or across multiple callsets. Similar to other SV merging tools (Jeffares *et al.* 2017, English *et al.* 2022, Kirsche *et al.* 2023), Verix computes the pairwise agreement between input records. For Verix, each record is an assembled CSV event, and agreement is defined as a full breakpoint alignment—namely, when every breakpoint in one event is matched to a distinct breakpoint in the other within the user-specified distance threshold (Fig. 1C). This harmonization process is formulated as a search for connected components (CSV clusters) in a CSV consensus graph, where each input CSV is represented as a node, and an edge is added between two CSV nodes iff all their breakpoints are concordant.

For each identified CSV cluster, Verix selects a primary representative to create a single consensus VCF record. To preserve provenance, every consensus record includes information about all the CSV events merged into it (e.g. their SV ID, type, and breakpoints). Similar to other SV consensus tools, each record also includes the following attributes for downstream analysis: (i) a support count indicating the number of input callsets contributing CSV(s) to this cluster, (ii) support count vector providing the total number of records merged in this cluster from each input VCF (since multiple events from the same VCF can be near-duplicates), and (iii) a binary support pattern encoding callset presence/absence.

### 2.3 Input requirements and outputs

Verix accepts VCF files as input and supports several common conventions for representing multi-breakpoint CSVs. In its default mode, two VCF records are linked via the standard BND mate notation. For events spanning more than two records, Verix supports two grouping modes: a *multi*-record mode, where all records sharing a common ID are aggregated into a single CSV; and a *single*-record mode, in which the breakpoints of a CSV are provided in a custom VCF INFO field. These formats can be specified independently for each input VCF, enabling direct comparisons across callsets produced by callers with different reporting conventions.

Once grouped, breakpoints from the same input CSV that fall within a configurable small distance are consolidated into a single breakpoint, allowing Verix to de-duplicate imprecise or redundant breakend reporting. Optional size filters may also be applied to exclude events with any inter-breakpoint intervals falling outside a specified range.

In benchmarking mode, Verix produces (i) a fully annotated VCF of the query callset, where each record carries its match classification, optimal ground-truth match, all candidate alignments considered, and relevant diagnostic flags, (ii) the assembled CSV representations of the query and target inputs as standalone VCFs, enabling inspection of the parsed events independently of matching results, (iii) a JSON report containing an extensive set of benchmarking metrics, and (iv) an optional PDF report with diagnostic plots. The harmonization command similarly produces an annotated consensus VCF and a JSON summary of cluster-level statistics.

### 2.4 Case study

To illustrate the use of Verix for CSV evaluation, we benchmarked three recent complex SV callers—SVision (v1.4) (Lin *et al.* 2022b), Sniffles2 (v2.6.3) (Smolka *et al.* 2024), and Severus (v1.6) (Keskus *et al.* 2026)—on a simulated PacBio HiFi long-read dataset. These callers report CSVs using different conventions, all of which Verix supports out of the box.

We generated a synthetic SV truthset using insilicoSV (v0.1.9) (Battistella *et al.* 2025), comprising five SV classes with different numbers of breakpoints and breakpoint types: simple deletions (DEL), inverted duplications (DUP_INV), dispersed duplications (dDUP), and inversions flanked by a deletion on one (INVdel) or both (delINVdel) sides. SV segment sizes were sampled uniformly from 1000 to 10 000 bp and dispersion distances from 50 000 to 100 000 bp; 200 instances of each SV type were placed uniformly at random across chromosome one of GRCh38. PacBio HiFi reads were simulated using PBSIM3 (Ono *et al.* 2022) (v3.0.5) at 30× coverage and aligned to GRCh38 using minimap2 (Li 2018) (v2.28). To ensure reproducibility, insilicoSV and PBSIM3 were run with a fixed seed of eighty-two.

Each caller was executed with its default parameters, and Verix was run with default settings when evaluating all callers (note: SV type and genotype matching are not enabled by default; δ is set to 500 bp by default for consistency with Truvari). The following configuration was used to parse each input VCF: *multi*-record with CLUSTERID INFO field for Severus, *single*-record with BKPS INFO field for SVision, and *default* for Sniffles2.

Benchmarking against the SV truthset revealed distinct reconstruction profiles across the three callers. Figure 1D and Tables 1–3, available as supplementary data at *Bioinformatics* online, show the summary statistics reported by Verix for each method, including the standard precision, recall, and F1 metrics (computed for *complete* matches) and match results stratified by category and SV type. Given the 1000 ground-truth SVs, the callers emitted 2011 (SVision), 1642 (Sniffles2), and 875 (Severus) events. While all three tools achieved comparable complete-match recall (SVision 48.5%, Sniffles2 39.6%, and Severus 40.3%), Severus achieved the highest precision (64.3%).

Stratifying by target SV type shows that most of the complete matches involve the two-breakpoint events (DEL and DUP_INV). In particular, all three callers recovered ≥99% of DELs (shown in the complete-match target_type_counts panel), and both Sniffles2 and Severus matched >90% of DUP_INVs (though these were emitted as separate simple DUP and INV records, shown in the complete-match query_type_counts panel). Recovery of the higher-cardinality classes (dDUP, INVdel, and delINVdel) was substantially lower: SVision generated the largest number of complete matches for INVdels (13%) and delINVdels (63.5%), while Severus found 3% of INVdels and 1.5% of delINVdels. These classes were instead primarily captured as partial matches, indicating that their breakpoints were detected but not assembled into single CSV events. Notably, Severus was the only caller to produce aggregate matches, showing that some of its records combined breakpoints from multiple distinct truth events.

For SVision and Sniffles2, Fig. 1D also shows the predicted-versus-true SV type correspondence heatmap, which can be especially informative for diagnosing partial matches, where simple events are frequently reported for subsets of CSV breakpoints. Because some CSV types decompose into multiple non-overlapping simple events (e.g. a delINVdel could be reported as a simple DEL, INV, and DEL), for each optimal target match Verix additionally reports whether the matched target breakpoints are consecutive (the count of such matches is shown in parentheses in each heatmap cell). For example, of the 190 INVs predicted by SVision that partially matched delINVdels, only two matched to consecutive breakpoints of target CSVs (likely corresponding to the INV segment), while none of the 254 INVs predicted by Sniffles2 match the INV segment of the delINVdel (notably, a large number of its partial INVs are also fragmented).

To illustrate Verix’s harmonization capability, we generated a consensus callset from the three caller outputs analyzed above, comprising a total of 4528 input records, which Verix collapsed into 2729 consensus clusters, a 39.7% reduction in callset size (Table 4, available as supplementary data at *Bioinformatics* online). Of these clusters, 1820 were supported by a single caller (including 1813 singletons), 909 were supported by at least two callers, and 505 were supported by all three. The most common multi-caller support pattern was full concordance with one record per caller (support vector 1,1,1 with *n* = 378 clusters); caller-private singletons were dominated by SVision (*n* = 1176) and Sniffles2 (*n* = 544), consistent with their lower precision in the benchmarking analysis. The largest clusters collapsed seven CSVs; e.g. one of them comprised multiple near-duplicate predictions of differing SV type from each caller at a DUP_INV site.

Across all analyses in the case study, Verix’s runtime was negligible (∼ 1 s). To further assess the computational scalability of Verix, we evaluated its runtime and peak memory usage across a wide range of CSV complexities (ranging from 2 to 128 breakpoints per CSV). We found that even in the most complex scenario, comparing VCFs with 1000 CSVs of 128 breakpoints each (which corresponds to callsets with a total of 128 000 genomic breakpoints), Verix completed the comparison in 3.9 minutes while requiring only 265 MB of RAM (Table 5, available as supplementary data at *Bioinformatics* online).

## Supplementary Material

btag803_Supplementary_Data

## References

[btag803-B1] Battistella E , Jiang N, Rohlicek C et al Insilicosv: a flexible grammar-based framework for structural variant simulation and placement. Bioinformatics 2025;41:btaf594. 10.1093/bioinformatics/btaf594 PMC12603351 41172269

[btag803-B2] Collins RL , Brand H, Karczewski KJ et al A structural variation reference for medical and population genetics. Nature 2020;581:444–51. 10.1038/s41586-020-2287-8 PMC7334194 32461652

[btag803-B3] Cosenza MR , Rodriguez-Martin B, Korbel JO et al Structural variation in cancer: role, prevalence, and mechanisms. Annu Rev Genomics Hum Genet 2022;23:123–52. 10.1146/annurev-genom-120121-101149 35655332

[btag803-B4] Ek M , Kvarnung M, Ten Berk de Boer E et al Long-read genome sequencing enhances diagnostics of pediatric neurological disorders. Genome Med 2026;18:12. 10.1186/s13073-025-01596-5 PMC12838436 41514368

[btag803-B5] English AC , Menon VK, Gibbs RA et al Truvari: refined structural variant comparison preserves allelic diversity. Genome Biol 2022;23:271. 10.1186/s13059-022-02840-6 PMC9793516 36575487

[btag803-B6] Heller D , Vingron M. SVIM: structural variant identification using mapped long reads. Bioinformatics 2019;35:2907–15. 10.1093/bioinformatics/btz041 PMC6735718 30668829

[btag803-B7] Jeffares DC , Jolly C, Hoti M et al Transient structural variations have strong effects on quantitative traits and reproductive isolation in fission yeast. Nat Commun 2017;8:14061. 10.1038/ncomms14061 PMC5286201 28117401

[btag803-B8] Jung H , Yang T-P, Walker S et al Complex de novo structural variants are an underestimated cause of rare disorders. Nat Commun 2025;16:9528. 10.1038/s41467-025-64722-2 PMC12583596 41184278

[btag803-B9] Keskus AG , Bryant A, Ahmad T et al Severus detects somatic structural variation and complex rearrangements in cancer genomes using long-read sequencing. Nat Biotechnol 2026;44:247–57. 10.1038/s41587-025-02618-8 PMC12483193 40185952

[btag803-B10] Kirsche M , Prabhu G, Sherman R et al Jasmine and Iris: population-scale structural variant comparison and analysis. Nat Methods 2023;20:408–17. 10.1038/s41592-022-01753-3 PMC10006329 36658279

[btag803-B11] Li H. Minimap2: pairwise alignment for nucleotide sequences. Bioinformatics 2018;34:3094–100. 10.1093/bioinformatics/bty191 PMC6137996 29750242

[btag803-B12] Li Y , Roberts ND, Wala JA et al Patterns of somatic structural variation in human cancer genomes. Nature 2020;578:112–21. 10.1038/s41586-019-1913-9 PMC7025897 32025012

[btag803-B13] Lin J , Wang S, Audano PA et al SVision: a deep learning approach to resolve complex structural variants. Nat Methods 2022a;19:1230–3. 10.1038/s41592-022-01609-w PMC9985066 36109679

[btag803-B14] Lin J , Yang X, Kosters W et al Mako: a graph-based pattern growth approach to detect complex structural variants. Genom Proteom Bioinform 2022b;20:205–18. 10.1016/j.gpb.2021.03.007 PMC9510932 34224879

[btag803-B15] Mantere T , Kersten S, Hoischen A et al Long-read sequencing emerging in medical genetics. Front Genet 2019;10:426. 10.3389/fgene.2019.00426 PMC6514244 31134132

[btag803-B16] Ono Y , Hamada M, Asai K et al PBSIM3: a simulator for all types of PACBIO and ONT long reads. NAR Genom Bioinform 2022;4:lqac092. 10.1093/nargab/lqac092 PMC9713900 36465498

[btag803-B17] Popic V , Rohlicek C, Cunial F et al Cue: a deep-learning framework for structural variant discovery and genotyping. Nat Methods 2023;20:559–68. 10.1038/s41592-023-01799-x PMC10152467 36959322

[btag803-B18] Sedlazeck FJ , Rescheneder P, Smolka M et al Accurate detection of complex structural variations using single-molecule sequencing. Nat Methods 2018;15:461–8. 10.1038/s41592-018-0001-7 PMC5990442 29713083

[btag803-B19] Smolka M , Paulin LF, Grochowski CM et al Detection of mosaic and population-level structural variants with sniffles2. Nat Biotechnol 2024;42:1571–80. 10.1038/s41587-023-02024-y PMC11217151 38168980

[btag803-B20] Soylev A , Le TM, Amini H et al Discovery of tandem and interspersed segmental duplications using high-throughput sequencing. Bioinformatics 2019;35:3923–30. 10.1093/bioinformatics/btz237 PMC6792081 30937433

