# Supplementary material for: Verix: a toolkit for benchmarking and harmonization of complex structural variants
Source: Bioinformatics. 2026 Oct 29;42(11):btag803. doi: 10.1093/bioinformatics/btag803 (PMC13755762; doi:10.1093/bioinformatics/btag803)
Supplement: Supplementary file 1 [file btag803_supplementary_data.pdf]

Supplementary Data

Supplementary Table 1: **Verix benchmarking, SVision**

Callset-level summary	
n_query	2,011
n_target	1,000
tp_query	502
tp_target	485
fp	1,509
fn	515
precision	0.250
recall	0.485
f1	0.330
class_proportions	
complete	0.250
partial	0.676
aggregate	0.000
spurious	0.075
complete (num_matches = 502)	
num_unique_targets	485
num_contiguous_matches	502
mean_breakpoint_distance	19.59
mean_breakpoint_hit_rate	1.000
mean_spurious_breakpoint_rate	0.000
mean_targets_per_record	1.000
complete: num_matches_by_query_target_type (num_contiguous)	
DEL/DEL	199 (199)
DEL+INV/delINVdel	127 (127)
tDUP/DUP_INV	91 (91)
DEL+INV/INVdel	26 (26)
INS/DUP_INV	22 (22)
INV+tDUP/DUP_INV	15 (15)
INV/DUP_INV	13 (13)
INS+INV/DUP_INV	5 (5)
INS+tDUP/DUP_INV	4 (4)
complete: query_type_counts	
DEL	199 (0.382)
DEL+INV	153 (0.677)
tDUP	91 (0.410)
INS	22 (0.118)
INV+tDUP	15 (0.484)
INV	13 (0.023)
INS+INV	5 (0.064)
INS+tDUP	4 (0.308)
complete: target_type_counts	
DEL	199 (0.995)
DUP_INV	133 (0.665)
delINVdel	127 (0.635)
INVdel	26 (0.130)

Supplementary Table 1: **Verix benchmarking, SVision (continued)**

partial (`num_matches` = 1,359)	
`num_unique_targets`	683
`num_contiguous_matches`	305
`mean_breakpoint_distance`	20.08
`mean_breakpoint_hit_rate`	0.510
`mean_spurious_breakpoint_rate`	0.252
`mean_targets_per_record`	1.000
partial: num_matches_by_query_target_type (num_contiguous)	
INV/INVdel	205 (13)
INV/delINVdel	190 (2)
INS/dDUP	164 (0)
DEL/dDUP	146 (145)
tDUP/dDUP	131 (0)
DEL/delINVdel	103 (0)
INV+DUP/DUP_INV	90 (0)
INS+INV/DUP_INV	73 (73)
DEL+INV/INVdel	72 (72)
DEL/INVdel	66 (0)
INV+DUP/delINVdel	42 (0)
INV/DUP_INV	29 (0)
INV+DUP/INVdel	18 (0)
INV+tDUP/DUP_INV	16 (0)
INS+tDUP/dDUP	9 (0)
DEL/DEL	2 (0)
DEL/DUP_INV	2 (0)
DEL+INV/delINVdel	1 (0)
partial: query_type_counts	
INV	424 (0.739)
DEL	319 (0.612)
INS	164 (0.882)
INV+DUP	150 (0.938)
tDUP	131 (0.590)
DEL+INV	73 (0.323)
INS+INV	73 (0.936)
INV+tDUP	16 (0.516)
INS+tDUP	9 (0.692)
partial: target_type_counts	
dDUP	197 (0.985)
INVdel	176 (0.880)
delINVdel	169 (0.845)
DUP_INV	139 (0.695)
DEL	2 (0.010)

Supplementary Table 2: **Verix benchmarking, Sniffles2**

Callset-level summary	
n_query	1,642
n_target	1,000
tp_query	572
tp_target	396
fp	1,070
fn	604
precision	0.348
recall	0.396
f1	0.371
class_proportions	
complete	0.348
partial	0.650
aggregate	0.000
spurious	0.001
complete (num_matches = 572)	
num_unique_targets	396
num_contiguous_matches	572
mean_breakpoint_distance	1.32
mean_breakpoint_hit_rate	1.000
mean_spurious_breakpoint_rate	0.000
mean_targets_per_record	1.000
complete: num_matches_by_query_target_type (num_contiguous)	
DEL/DEL	198 (198)
INV/DUP_INV	197 (197)
DUP/DUP_INV	177 (177)
complete: query_type_counts	
DEL	198 (0.460)
INV	197 (0.271)
DUP	177 (0.717)
complete: target_type_counts	
DEL	198 (0.990)
DUP_INV	198 (0.990)

Supplementary Table 2: **Verix benchmarking, Sniffles2 (continued)**

partial (num_matches = 1,068)	
num_unique_targets	606
num_contiguous_matches	225
mean_breakpoint_distance	6.07
mean_breakpoint_hit_rate	0.543
mean_spurious_breakpoint_rate	0.001
mean_targets_per_record	1.000
partial: num_matches_by_query_target_type (num_contiguous)	
INV/INVdel	274 (171)
INV/delINVdel	254 (0)
INS/dDUP	193 (0)
DEL/delINVdel	102 (0)
DEL/INVdel	74 (0)
DUP/dDUP	70 (0)
DEL/dDUP	54 (54)
INS/DUP_INV	42 (0)
INS/delINVdel	3 (0)
DEL/DEL	1 (0)
DEL/DUP_INV	1 (0)
partial: query_type_counts	
INV	528 (0.727)
INS	238 (0.996)
DEL	232 (0.540)
DUP	70 (0.283)
partial: target_type_counts	
dDUP	197 (0.985)
INVdel	183 (0.915)
delINVdel	183 (0.915)
DUP_INV	42 (0.210)
DEL	1 (0.005)

Supplementary Table 3: **Verix benchmarking, Severus**

Callset-level summary	
`n_query`	875
`n_target`	1,000
`tp_query`	563
`tp_target`	403
`fp`	312
`fn`	597
`precision`	0.643
`recall`	0.403
`f1`	0.496
class_proportions	
`complete`	0.643
`partial`	0.262
`aggregate`	0.093
`spurious`	0.002
complete (`num_matches` = 563)	
`num_unique_targets`	403
`num_contiguous_matches`	563
`mean_breakpoint_distance`	1.22
`mean_breakpoint_hit_rate`	1.000
`mean_spurious_breakpoint_rate`	0.000
`mean_targets_per_record`	1.000
complete: num_matches_by_query_target_type (num_contiguous)	
DEL/DEL	198 (198)
INV/DUP_INV	189 (189)
DUP/DUP_INV	164 (164)
BND/INVdel	6 (6)
BND/delINVdel	3 (3)
BND/DUP_INV	2 (2)
DEL+DUP/dDUP	1 (1)
complete: query_type_counts	
DEL	198 (0.896)
INV	189 (1.000)
DUP	164 (0.927)
BND	11 (0.306)
DEL+DUP	1 (0.333)
complete: target_type_counts	
DEL	198 (0.990)
DUP_INV	195 (0.975)
INVdel	6 (0.030)
delINVdel	3 (0.015)
dDUP	1 (0.005)

Supplementary Table 3: **Verix benchmarking, Severus (continued)**

partial (num_matches = 229)	
num_unique_targets	196
num_contiguous_matches	21
mean_breakpoint_distance	2.45
mean_breakpoint_hit_rate	0.386
mean_spurious_breakpoint_rate	0.004
mean_targets_per_record	1.000
partial: num_matches_by_query_target_type (num_contiguous)	
INS/dDUP	187 (0)
DEL/dDUP	20 (20)
DUP/dDUP	13 (0)
INS/delINVdel	2 (0)
INS/DEL	2 (0)
DEL/DUP_INV	2 (0)
INS/DUP_INV	1 (0)
BND/delINVdel	1 (0)
BND/INVdel	1 (1)
partial: query_type_counts	
INS	192 (0.995)
DEL	22 (0.100)
DUP	13 (0.073)
BND	2 (0.056)
partial: target_type_counts	
dDUP	189 (0.945)
DUP_INV	3 (0.015)
delINVdel	2 (0.010)
INVdel	1 (0.005)
DEL	1 (0.005)

Supplementary Table 3: **Verix benchmarking, Severus (continued)**

aggregate (num_matches = 81)	
num_unique_targets	81
num_contiguous_matches	81
mean_breakpoint_distance	0.73
mean_breakpoint_hit_rate	0.996
mean_spurious_breakpoint_rate	0.017
mean_targets_per_record	6.63
aggregate: num_matches_by_query_target_type (num_contiguous)	
BND+DEL/delINVdel	25 (25)
BND/delINVdel	22 (22)
BND+DEL+DUP/delINVdel	18 (18)
BND+DEL/INVdel	7 (7)
BND+DEL+DUP/INVdel	3 (3)
BND+DEL+DUP/dDUP	2 (2)
DEL+DUP/dDUP	2 (2)
BND/INVdel	1 (1)
BND+DEL/dDUP	1 (1)
aggregate: query_type_counts	
BND+DEL	33 (1.000)
BND	23 (0.639)
BND+DEL+DUP	23 (1.000)
DEL+DUP	2 (0.667)
aggregate: target_type_counts	
delINVdel	65 (0.325)
INVdel	11 (0.055)
dDUP	5 (0.025)

Supplementary Table 4: **Verix consensus generated for three CSV callsets (SVision, Sniffles2, and Severus).**

Input callsets	
n_total_variants	4,528
n_variants_in_sample: SVision	2,011
n_variants_in_sample: Sniffles2	1,642
n_variants_in_sample: Severus	875
Consensus clusters	
n_clusters	2,729
n_singleton_clusters	1,813
n_multi_caller_clusters	909
n_full_support_clusters	505
max_cluster_size	7
min_cluster_size	1
support_vec_counts (SVision, Sniffles2, Severus)	
(1, 0, 0)	1,176
(0, 1, 0)	544
(1, 1, 1)	378
(1, 1, 0)	264
(1, 2, 2)	100
(0, 0, 1)	93
(0, 2, 2)	41
(0, 1, 1)	39
(1, 2, 0)	23
(1, 0, 1)	23
(2, 2, 2)	13
(0, 2, 1)	11
(1, 2, 1)	10
(0, 2, 0)	6
(0, 1, 2)	3
(3, 2, 2)	2
(2, 1, 1)	1
(2, 0, 0)	1
(1, 1, 2)	1
cluster_size_counts	
1	1,813
2	333
3	415
4	53
5	100
6	13
7	2

Supplementary Table 5: **Runtime and peak memory evaluation.** Verix was evaluated across seven generated target VCF files, each containing 1,000 CSVs with varying breakpoint counts ($n = 2, 4, 8, 16, 32, 64, \text{and } 128$). Each CSV was constructed from $n/2$ VCF records representing $n/2$ 1,000 bp segments spaced 200 bp apart (the distance between CSVs was set to 50,000 bp). A corresponding query VCF was generated for each target VCF by independently shifting each breakpoint coordinate by a random offset drawn from -50 to 50 bp. Verix was executed in benchmarking mode with default parameters ($\delta = 500$) and `--merge_thr=0` on each query-target VCF pair. Runtime and peak memory (maximum resident set size) were measured using a single thread on an Intel Xeon Gold 6258R system with 2TB of RAM.

Breakpoints per CSV (n)	Breakpoints per callset	Time (s)	Peak Memory (MB)
2	2,000	1.47	130.41
4	4,000	1.84	132.07
8	8,000	3.45	137.40
16	16,000	8.03	145.30
32	32,000	22.44	165.75
64	64,000	72.21	205.84
128	128,000	234.37	264.89
